# An integrative review on methodological considerations in mental health research – design, sampling, data collection procedure and quality assurance

**DOI:** 10.1186/s13690-019-0363-z

**Published:** 2019-10-10

**Authors:** Eric Badu, Anthony Paul O’Brien, Rebecca Mitchell

**Affiliations:** 1 School of Nursing and Midwifery, The University of Newcastle, Callaghan, Australia; 2 Faculty of Health and Medicine, School Nursing and Midwifery, University of Newcastle, Callaghan, Australia; 3 Faculty of Business and Economics, Macquarie University, North Ryde, Australia

**Keywords:** Mental health, Methodological approach, Mixed methods, Sampling, Data collection

## Abstract

**Background:**

Several typologies and guidelines are available to address the methodological and practical considerations required in mental health research. However, few studies have actually attempted to systematically identify and synthesise these considerations. This paper provides an integrative review that identifies and synthesises the available research evidence on mental health research methodological considerations.

**Methods:**

A search of the published literature was conducted using EMBASE, Medline, PsycINFO, CINAHL, Web of Science, and Scopus. The search was limited to papers published in English for the timeframe 2000–2018. Using pre-defined inclusion and exclusion criteria, three reviewers independently screened the retrieved papers. A data extraction form was used to extract data from the included papers.

**Results:**

Of 27 papers meeting the inclusion criteria, 13 focused on qualitative research, 8 mixed methods and 6 papers focused on quantitative methodology. A total of 14 papers targeted global mental health research, with 2 papers each describing studies in Germany, Sweden and China. The review identified several methodological considerations relating to study design, methods, data collection, and quality assurance. Methodological issues regarding the study design included assembling team members, familiarisation and sharing information on the topic, and seeking the contribution of team members. Methodological considerations to facilitate data collection involved adequate preparation prior to fieldwork, appropriateness and adequacy of the sampling and data collection approach, selection of consumers, the social or cultural context, practical and organisational skills; and ethical and sensitivity issues.

**Conclusion:**

The evidence confirms that studies on methodological considerations in conducting mental health research largely focus on qualitative studies in a transcultural setting, as well as recommendations derived from multi-site surveys. Mental health research should adequately consider the methodological issues around study design, sampling, data collection procedures and quality assurance in order to maintain the quality of data collection.

**Electronic supplementary material:**

The online version of this article (10.1186/s13690-019-0363-z) contains supplementary material, which is available to authorized users.

## Background

In the past decades there has been considerable attention on research methods to facilitate studies in various academic fields, such as public health, education, humanities, behavioural and social sciences [1–4]. These research methodologies have generally focused on the two major research pillars known as quantitative or qualitative research. In recent years, researchers conducting mental health research appear to be either employing both qualitative and quantitative research methods separately, or mixed methods approaches to triangulate and validate findings [5, 6].

A combination of study designs has been utilised to answer research questions associated with mental health services and consumer outcomes [7, 8]. Study designs in the public health and clinical domains, for example, have largely focused on observational studies (non-interventional) and experimental research (interventional) [1, 3, 9]. Observational design in non-interventional research requires the investigator to simply observe, record, classify, count and analyse the data [1, 2, 10]. This design is different from the observational approaches used in social science research, which may involve observing (participant and non- participant) phenomena in the fieldwork [1]. Furthermore, the observational study has been categorized into five types, namely cross-sectional design, case-control studies, cohort studies, case report and case series studies [1–3, 9–11]. The cross-sectional design is used to measure the occurrence of a condition at a one-time point, sometimes referred to as a prevalence study. This approach of conducting research is relatively quick and easy but does not permit a distinction between cause and effect [1]. Conversely, the case-control is a design that examines the relationship between an attribute and a disease by comparing those with and without the disease [1, 2, 12]. In addition, the case-control design is usually retrospective and aims to identify predictors of a particular outcome. This type of design is relevant when investigating rare or chronic diseases which may result from long-term exposure to particular risk factors [10]. Cohort studies measure the relationship between exposure to a factor and the probability of the occurrence of a disease [1, 10]. In a case series design, medical records are reviewed for exposure to determinants of disease and outcomes. More importantly, case series and case reports are often used as preliminary research to provide information on key clinical issues [12].

The interventional study design describes a research approach that applies clinical care to evaluate treatment effects on outcomes [13]. Several previous studies have explained the various forms of experimental study design used in public health and clinical research [14, 15]. In particular, experimental studies have been categorized into randomized controlled trials (RCTs), non-randomized controlled trials, and quasi-experimental designs [14]. The randomized trial is a comparative study where participants are randomly assigned to one of two groups. This research examines a comparison between a group receiving treatment and a control group receiving treatment as usual or receiving a placebo. Herein, the exposure to the intervention is determined by random allocation [16, 17].

Recently, research methodologists have given considerable attention to the development of methodologies to conduct research in vulnerable populations. Vulnerable population research, such as with mental health consumers often involves considering the challenges associated with sampling (selecting marginalized participants), collecting data and analysing it, as well as research engagement. Consequently, several empirical studies have been undertaken to document the methodological issues and challenges in research involving marginalized populations. In particular, these studies largely addresses the typologies and practical guidelines for conducting empirical studies in mental health. Despite the increasing evidence, however, only a few studies have yet attempted to systematically identify and synthesise the methodological considerations in conducting mental health research from the perspective of consumers.

A preliminary search using the search engines Medline, Web of Science, Google Scholar, and Scopus Index and EMBASE identified only two reviews of mental health based research. Among these two papers, one focused on the various types of mixed methods used in mental health research [18], whilst the other paper, focused on the role of qualitative studies in mental health research involving mixed methods [19]. Even though the latter two studies attempted to systematically review mixed methods mental health research, this integrative review is unique, as it collectively synthesises the design, data collection, sampling, and quality assurance issues together, which has not been previously attempted.

This paper provides an integrative review addressing the available evidence on mental health research methodological considerations. The paper also synthesises evidence on the methods, study designs, data collection procedures, analyses and quality assurance measures. Identifying and synthesising evidence on the conduct of mental health research has relevance to clinicians and academic researchers where the evidence provides a guide regarding the methodological issues involved when conducting research in the mental health domain. Additionally, the synthesis can inform clinicians and academia about the gaps in the literature related to methodological considerations.

## Methods

### Methodology

An integrative review was conducted to synthesise the available evidence on mental health research methodological considerations. To guide the review, the World Health Organization (WHO) definition of mental health has been utilised. The WHO defines mental health as: “a state of well-being, in which the individual realises his or her own potentials, ability to cope with the normal stresses of life, functionality and work productivity, as well as the ability to contribute effectively in community life” [20]. The integrative review enabled the simultaneous inclusion of diverse methodologies (i.e., experimental and non-experimental research) and varied perspectives to fully understand a phenomenon of concern [21, 22]. The review also uses diverse data sources to develop a holistic understanding of methodological considerations in mental health research. The methodology employed involves five stages: 1) problem identification (ensuring that the research question and purpose are clearly defined); 2) literature search (incorporating a comprehensive search strategy); 3) data evaluation; 4) data analysis (data reduction, display, comparison and conclusions) and; 5) presentation (synthesising findings in a model or theory and describing the implications for practice, policy and further research) [21].

### Inclusion criteria

The integrative review focused on methodological issues in mental health research. This included core areas such as study design and methods, particularly qualitative, quantitative or both. The review targeted papers that addressed study design, sampling, data collection procedures, quality assurance and the data analysis process. More specifically, the included papers addressed methodological issues on empirical studies in mental health research. The methodological issues in this context are not limited to a particular mental illness. Studies that met the inclusion criteria were peer-reviewed articles published in the English Language, from January 2000 to July 2018.

### Exclusion criteria

Articles that were excluded were based purely on general health services or clinical effectiveness of a particular intervention with no connection to mental health research. Articles were also excluded when it addresses non-methodological issues. Other general exclusion criteria were book chapters, conference abstracts, papers that present opinion, editorials, commentaries and clinical case reviews.

### Search strategy and selection procedure

The search of published articles was conducted from six electronic databases, namely EMBASE, CINAHL (EBSCO), Web of Science, Scopus, PsycINFO and Medline. We developed a search strategy based on the recommended guidelines by the Joanna Briggs Institute (JBI) [23]. Specifically, a three-step search strategy was utilised to conduct the search for information (see Table 1). An initial limited search was conducted in Medline and Embase (see Table 1). We analysed the text words contained in the title and abstract and of the index terms from the initial search results [23]. A second search using all identified keywords and index terms was then repeated across all remaining five databases (see Table 1). Finally, the reference lists of all eligible studies were manually hand searched [23].

**Table 1:** Search strategy and selection procedure

Stages	Search terms and keywords
Stage 1 (Initial search in MEDLINE and EMBASE	(“mental health” OR mental health service OR “psychiatric services” OR mental disorders OR mental illness) AND (“methods” or “research designs” or “data collection” or “data analysis” OR “sampling” or “sample size” OR “mixed methods”) AND (“quality assurance” OR “reliability” OR “validity” OR “techniques” OR “strategies” OR research design OR “informed consent”)
Stage 2 (search across CINAHL, Web of Science, Scopus, and PsycINFO)	(“psychiatry” OR “mental health” OR “mental disorders” OR “mental patient” OR “mental illness” OR “mental treatment” OR “consumer”) AND (“research methods” OR “methodology” OR “research designs” OR “qualitative research” OR “quantitative research” OR “mixed methods” OR “biomedical research” OR “health service research” OR “epidemiologic methods” OR “behavioural research” OR “process design”) AND (“sampling” OR “sample size” OR “patient selection” OR “surveys” OR “questionnaires” OR “interviews” OR “data analysis” OR “content analysis” OR “thematic analysis” OR “reporting”) AND (“informed consent” “reliability” OR “quality assurance” OR “validity” OR “techniques” OR “strategies” OR “process”)
Stage 3	Hand searching of the reference lists

**Fig. 1:**
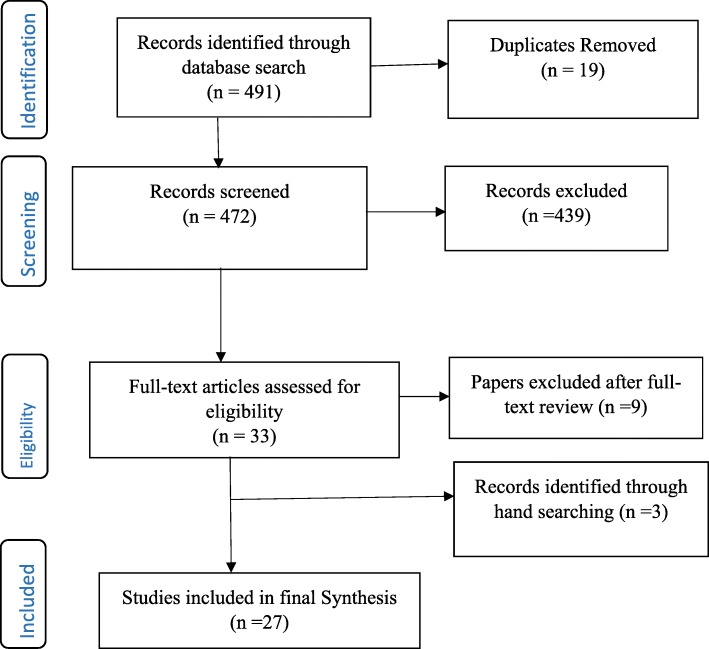
Flow Chart of studies included in the review

The selection of eligible articles adhered to the Preferred Reporting Items for Systematic Reviews and Meta-Analyses (PRISMA) [24] (see Fig. 1). Firstly, three authors independently screened the titles of articles that were retrieved and then approved those meeting the selection criteria. The authors reviewed all the titles and abstracts and agreed on those needing full-text screening. E.B (Eric Badu) conducted the initial screening of titles and abstracts. A.P.O’B (Anthony Paul O’Brien) and R.M (Rebecca Mitchell) conducted the second screening of titles and abstracts of all the identified papers. The authors (E.B, A.P.O’B and R.M) conducted full-text screening according to the inclusion and exclusion criteria.

### Data management and extraction

The integrative review used Endnote ×8 to screen and handle duplicate references. A predefined data extraction form was developed to extract data from all included articles (see Additional file 1). The data extraction form was developed according to Joanna Briggs Institute (JBI) [23] and Cochrane [24] manuals, as well as the literature associated with concepts and methods in mental health research. The data extraction form was categorised into sub-sections, such as study details (citation, year of publication, author, contact details of lead author, and funder/sponsoring organisation, publication type), objective of the paper, primary subject area of the paper (study design, methods, sampling, data collection, data analysis, quality assurance). The data extraction form also had a section on additional information on methodological consideration, recommendations and other potential references. The authors extracted results of the included papers in numerical and textual format [23]. EB (Eric Badu) conducted the data extraction, A.P.O’B (Anthony Paul O’Brien) and R.M (Rebecca Mitchell), conducted the second review of the extracted data.

### Data synthesis

Content analysis was used to synthesise the extracted data. The content analysis process involved several stages which involved noting patterns and themes, seeing plausibility, clustering, counting, making contrasts and comparisons, discerning common and unusual patterns, subsuming particulars into general, noting relations between variability, finding intervening factors and building a logical chain of evidence [21] (see Table 2).

**Table 2:** The key emerging themes

Theme	Sub-theme	N^a^	Papers
Mixed methods design in mental health research	Categorizing mixed methods	4	(19) (18) (43) (48)
	Function of mixed methods	6	(45) (42) (48) (19) (18) (43)
	Structure of mixed methods	5	(43) (19) (18) (42) (48)
	Process of mixed methods	5	(48) (43) (42) (19) (18)
	Consideration for using mixed methods	3	(19) (18) (45)
Qualitative study in mental health research	Considering qualitative methods	6	(32) (36) (19) (26) (28) (44)
Sampling in mental health research	Sampling approaches (quantitative)	3	(35) (34) (25)
	Sampling approaches (qualitative)	7	(28) (32) (46) (19) (42) (30) (31)
	Sampling consideration	4	(30) (31) (32) (46)
Data collection in mental health research	Approaches for collecting qualitative data	9	(28) (41) (30) (31) (44) (47) (19) (40) (34)
	Consideration for data collection	6	(32) (37) (31) (41) (49) (47)
	Preparing for data collection	8	(25) (33) (34) (35) (39) (41) (49) (30)
Quality assurance procedures	Seeking informed consent	7	(25) (26) (33) (35) (37) (39) (47)
	Procedure for ensuring quality control (quantitative)	5	(49) (25) (39) (33) (38)
	Procedure for ensuring quality control (qualitative)	4	(32) (37) (46) (19)

N^a^ number of papers

## Results

### Study characteristics

The integrative review identified a total of 491 records from all databases, after which 19 duplicates were removed. Out of this, 472 titles and abstracts were assessed for eligibility, after which 439 articles were excluded. Articles not meeting the inclusion criteria were excluded. Specifically, papers excluded were those that did not address methodological issues as well as papers addressing methodological consideration in other disciplines. A total of 33 full-text articles were assessed – 9 articles were further excluded, whilst an additional 3 articles were identified from reference lists. Overall, 27 articles were included in the final synthesis (see Fig. 1). Of the total included papers, 12 contained qualitative research, 9 were mixed methods (both qualitative and quantitative) and 6 papers focused on quantitative data. Conversely, a total of 14 papers targeted global mental health research and 2 papers each describing studies in Germany, Sweden and China. The papers addressed different methodological issues, such as study design, methods, data collection, and analysis as well as quality assurance (see Table 3).

**Table 3:** Study characteristics

Author	Setting	Methodological issues addressed	Type of method
Alonso, Angermeyer [25]	Belgium, France, Germany, Italy, the Netherlands and Spain	Sampling, data collection and Quality assurance	Quantitative
Baarnhielm and Ekblad [26]	Sweden	Quality assurance (ethical issues)	Qualitative
Braun and Clarke [27]	Global	Data analysis	Qualitative
Brown and Lloyd [28]	Global	Methods, sampling, data collection and analysis	Qualitative
Davidsen [29]	Global	Data analysis	Qualitative
de Jong and Van Ommeren [30]	Global	Sampling and Data collection	Mixed Methods
Ekblad and Baarnhielm [31]	Sweden	Data collection	Qualitative
Fossey, Harvey [32]	Global	Methods, Sampling, data collection, data analysis and Quality assurance	Qualitative
Jacobi, Wittchen [33]	Germany	Data collection, analysis and Quality assurance	Quantitative
Koch, Vogel [34]	Germany	Sampling, data collection and Quality assurance	Mixed Methods
Korver, Quee [35]	Netherlands	Sampling and Quality assurance	Quantitative
Larkin, Watts [36]	Global	Study design	Qualitative
Latvala, Vuokila-Oikkonen [37]	Finland	Data collection and Quality assurance	Qualitative
Leese, White [38]	Europe	Quality assurance	Quantitative
Liu, Huang [39]	China	Data analysis and Quality assurance	Quantitative
Montgomery and Bailey [40]	Canada	Data collection and analysis	Qualitative
Owen [41]	UK	Data collection	Qualitative
Palinkas [19]	Global	Study design, methods, sampling, data collection, analysis and Quality assurance	Mixed Methods
Palinkas, Horwitz [18]	Global	Study design	Mixed Methods
Palinkas, Horwitz [42]	Global	Sampling	Mixed methods
Palinkas, Aarons [43]	Global	Study design	Mixed Methods
Razafsha, Behforuzi [44]	Global	Methods and data collection	Mixed Methods
Robins, Ware [45]	Global	Study design	Mixed Methods
Robinson [46]	Global	Sampling and Quality assurance	Qualitative
Schilder, Tomov [47]	Bulgaria	Data collection	Qualitative
Schoonenboom and Johnson [48]	Global	Study design	Mixed Methods
Yin, Phillips [49]	China	Data collection	Quantitative

### Mixed methods design in mental health research

Mixed methods research is defined as a research process where the elements of qualitative and quantitative research are combined in the design, data collection, and its triangulation and validation [48]. The integrative review identified four sub-themes that describe mixed methods design in the context of mental health research. The sub-themes include the categories of mixed methods, their function, structure, process and further methodological considerations for mixed methods design. These sub-themes are explained as follows:

### Categorizing mixed methods in mental health research

Four studies highlighted the categories of mixed methods design applicable to mental health research [18, 19, 43, 48]. Generally, there are differences in the categories of mixed methods design, however, three distinct categories predominantly appear to cross cut in all studies. These categories are function, structure and process. Some studies further categorised mixed method design to include rationale, objectives, or purpose. For instance, Schoonenboom and Johnson [48] categorised mixed methods design into primary and secondary dimensions.

### The function of mixed methods in mental health research

Six studies explain the function of conducting mixed methods design in mental health research. Two studies specifically recommended that mixed methods have the ability to provide a more robust understanding of services by expanding and strengthening the conclusions from the study [42, 45]. More importantly, the use of both qualitative and quantitative methods have the ability to provide innovative solutions to important and complex problems, especially by addressing diversity and divergence [48]. The review identified five underlying functions of a mixed method design in mental health research which include achieving convergence, complementarity, expansion, development and sampling [18, 19, 43].

The use of mixed methods to achieve convergence aims to employ both qualitative and quantitative data to answer the same question, either through triangulation (to confirm the conclusions from each of the methods) or transformation (using qualitative techniques to transform quantitative data). Similarly, complementarity in mixed methods integrates both qualitative and quantitative methods to answer questions for the purpose of evaluation or elaboration [18, 19, 43]. Two papers recommend that qualitative methods are used to provide the depth of understanding, whilst the quantitative methods provide a breadth of understanding [18, 43]. In mental health research, the qualitative data is often used to examine treatment processes, whilst the quantitative methods are used to examine treatment outcomes against quality care key performance targets.

Additionally, three papers indicated that expansion as a function of mixed methods uses one type of method to answer questions raised by the other type of method [18, 19, 43]. For instance, qualitative data is used to explain findings from quantitative analysis. Also, some studies highlight that development as a function of mixed methods aims to use one method to answer research questions, and use the findings to inform other methods to answer different research questions. A qualitative method, for example, is used to identify the content of items to be used in a quantitative study. This approach aims to use qualitative methods to create a conceptual framework for generating hypotheses to be tested by using a quantitative method [18, 19, 43]. Three papers suggested that using mixed methods for the purpose of sampling utilize one method (eg. quantitative) to identify a sample of participants to conduct research using other methods (eg. qualitative) [18, 19, 43]. For instance, quantitative data is sequentially utilized to identify potential participants to participate in a qualitative study and the vice versa.

### Structure of mixed methods in mental health research

Five studies categorised the structure of conducting mixed methods in mental health research, into two broader concepts including simultaneous (concurrent) and sequential (see Table 3). In both categories, one method is regarded as primary and the other as secondary, although equal weight can be given to both methods [18, 19, 42, 43, 48]. Two studies suggested that the sequential design is a process where the data collection and analysis of one component (eg. quantitative) takes place after the data collection and analysis of the other component (eg qualitative). Herein, the data collection and analysis of one component (e.g. qualitative) may depend on the outcomes of the other component (e.g. quantitative) [43, 48]. An earlier review suggested that the majority of contemporary studies in mental health research use a sequential design, with qualitative methods, more often preceding quantitative methods [18].

Alternatively, the concurrent design collects and analyses data of both components (e.g. quantitative and qualitative) simultaneously and independently. Palinkas, Horwitz [42] recommend that one component is used as secondary to the other component, or that both components are assigned equal priority. Such a mixed methods approach aims to provide a depth of understanding afforded by qualitative methods, with the breadth of understanding offered by the quantitative data to elaborate on the findings of one component or seek convergence through triangulation of the results. Schoonenboom and Johnson [48] recommended the use of capital letters for one component and lower case letters for another component in the same design to indicate that one component is primary and the other is secondary or supplemental.

### Process of mixed methods in mental health research

Five papers highlighted the process for the use of mixed methods in mental health research [18, 19, 42, 43, 48]. The papers suggested three distinct processes or strategies for combining qualitative and quantitative data. These include merging or converging the two data sets, connecting the two datasets by having one build upon the other; and embedding one data set within the other [19, 43]. The process of connecting occurs when the analysis of one dataset leads to the need for the other data set. For instance, in the situation where quantitative results lead to the subsequent collection and analysis of qualitative data [18, 43]. A previous study suggested that most studies in mental health sought to connect the data sets. Similarly, the process of merging the datasets brings together two sets of data during the interpretation, or transforms one type of data into the other type, by combining the data into new variables [18]. The process of embedding data into mixed method designs in mental health uses one dataset to provide a supportive role to the other dataset [43].

### Consideration for using mixed methods in mental health research

Three studies highlighted several factors that need to be considered when conducting mixed methods design in mental health research [18, 19, 45]. Accordingly, these factors include developing familiarity with the topic under investigation based on experience, willingness to share information on the topic [19], establishing early collaboration, willingness to negotiate emerging problems, seeking the contribution of team members, and soliciting third-party assistance to resolve any emerging problems [45]. Additionally, Palinkas, Horwitz [18] recommended that mixed methods in the context of mental health research are mostly applied in studies that assess needs of services, examine existing services, developing new or adapting existing services, evaluating services in randomised control trials, and examining service implementation.

### Qualitative study in mental health research

This theme describes the various qualitative methods used in mental health research. The theme also addresses methodological considerations for using qualitative methods in mental health research. The key emerging issues are discussed below:

### Considering qualitative components in conducting mental health research

Six studies recommended the use of qualitative methods in mental health research [19, 26, 28, 32, 36, 44]. Two qualitative research paradigms were identified, including the interpretive and critical approach [32]. The interpretive methodologies predominantly explore the meaning of human experiences and actions, whilst the critical approach emphasises the social and historical origins and contexts of meaning [32]. Two studies suggested that the interpretive qualitative methods used in mental health research are ethnography, phenomenology and narrative approaches [32, 36].

The ethnographic approach describes the everyday meaning of the phenomena within a societal and cultural context, for instance, the way phenomena or experience is contrasted within a community, or by collective members over time [32]. Alternatively, the phenomenological approach explores the claims and concerns of a subject with a speculative development of an interpretative account within their cultural and physical environments focusing on the lived experience [32, 36].

Moreover, the critical qualitative approaches used in mental health research are predominantly emancipatory (for instance, socio-political traditions) and participatory action-based research. The emancipatory traditions recognise that knowledge is acquired through critical discourse and debate but are not seen as discovered by objective inquiry [32]. Alternatively, the participatory action based approach uses critical perspectives to engage key stakeholders as participants in the design and conduct of the research [32].

Some studies highlighted several reasons why qualitative methods are relevant to mental health research. In particular, qualitative methods are significant as they emphasise naturalistic inquiry and have a discovery-oriented approach [19, 26]. Two studies suggested that qualitative methods are often relevant in the initial stages of research studies to understand specific issues such as behaviour, or symptoms of consumers of mental services [19]. Specifically, Palinkas [19] suggests that qualitative methods help to obtain initial pilot data, or when there is too little previous research or in the absence of a theory, such as provided in exploratory studies, or previously under-researched phenomena.

Three studies stressed that qualitative methods can help to better understand socially sensitive issues, such as exploring the solutions to overcome challenges in mental health clinical policies [19, 28, 44]. Consequently, Razafsha, Behforuzi [44] recommended that the natural holistic view of qualitative methods can help to understand the more recovery-oriented policy of mental health, rather than simply the treatment of symptoms. Similarly, the subjective experiences of consumers using qualitative approaches have been found useful to inform clinical policy development [28].

### Sampling in mental health research

The theme explains the sampling approaches used in mental health research. The section also describes the methodological considerations when sampling participants for mental health research. The sub-themes emerging are explained in the following sections:

### Sampling approaches (quantitative)

Some studies reviewed highlighted the sampling approaches previously used in mental health research [25, 34, 35]. Generally, all quantitative studies tend to use several probability sampling approaches, whilst qualitative studies used non-probability techniques. The quantitative mental health studies conducted at community and population level employ multi-stage sampling techniques usually involving systematic sampling, stratified and random sampling [25, 34]. Similarly, quantitative studies that recruit consumers in the hospital setting employ consecutive sampling [35]. Two studies reviewed highlighted that the identification of consumers of mental health services for research is usually conducted by service providers. For instance, Korver, Quee [35] research used a consecutive sampling approach by identifying consumers through clinicians working in regional psychosis departments, or academic centres.

### Sampling approaches (qualitative)

Seven studies suggested that the sampling procedures widely used in mental health research involving qualitative methods are non-probability techniques, which include purposive [19, 28, 32, 42, 46], snowballing [30, 32, 46] and theoretical sampling [31, 32]. The purposive sampling identifies participants that possess relevant characteristics to answer a research question [28]. Purposive sampling can be used in a single case study, or for multiple cases. The purposive sampling used in mental health research is usually extreme, or deviant case sampling, criterion sampling, and maximum variation sampling [19]. Furthermore, it is advised when using purposive sampling in a multistage level study, that it should aim to begin with the broader picture to achieve variation, or dispersion, before moving to the more focused view that considers similarity, or central tendencies [42].

Two studies added that theoretical sampling involved sampling participants, situations and processes based on concepts on theoretical grounds and then using the findings to build theory, such as in a Grounded Theory study [31, 32]. Some studies highlighted that snowball sampling is another strategy widely used in mental health research [30, 32, 46]. This is ascribed to the fact that people with mental illness are perceived as marginalised in research and practically hard-to-reach using conventional sampling [30, 32]. Snowballing sampling involves asking the marginalised participants to recommend individuals who might have direct knowledge relevant to the study [30, 32, 46]. Although this approach is relevant, some studies advise the limited possibility of generalising the sample, because of the likelihood of selection bias [30].

### Sampling consideration

Four studies in this section highlighted some of the sampling considerations in mental health research [30–32, 46]. Generally, mental health research should consider the appropriateness and adequacy of sampling approach by applying attributes such as shared social, or cultural experiences, or shared concern related to the study [32], diversity and variety of participants [31], practical and organisational skills, as well as ethical and sensitivity issues [46]. Robinson [46] further suggested that sampling can be homogenous or heterogeneous depending on the research questions for the study. Achieving homogeneity in sampling should employ a variety of parameters, which include demographic, graphical, physical, psychological, or life history homogeneity [46]. Additionally, applying homogeneity in sampling can be influenced by theoretical and practical factors. Alternatively, some samples are intentionally selected based on heterogeneous factors [46].

### Data collection in mental health research

This theme highlights the data collection methods used in mental health research. The theme is explained according to three sub-themes, which include approaches for collecting qualitative data, methodological considerations, as well as preparations for data collection. The sub-themes are as follows:

### Approaches for collecting qualitative data

The studies reviewed recommended the approaches that are widely applied in collecting data in mental health research. The widely used qualitative data collection approaches in mental health research are focus group discussions (FGDs) [19, 28, 30, 31, 41, 44, 47], extended in-depth interviews [19, 30, 34], participant and non-participant observation [19], Delphi data collection, quasi-statistical techniques [19] and field notes [31, 40]. Seven studies suggest that FGDs are widely used data collection approaches [19, 28, 30, 31, 41, 44, 47] because they are valuable in gathering information on consumers’ perspectives of services, especially regarding satisfaction, unmet/met service needs and the perceived impact of services [47]. Conversely, Ekblad and Baarnhielm [31] recommended that this approach is relevant to improve clinical understanding of the thoughts, emotions, meanings and attitudes towards mental health services.

Such data collection approaches are particularly relevant to consumers of mental health services, due to their low self-confidence and self-esteem [41]. The approach can help to understand specific terms, vocabulary, opinions and attitudes of consumers of mental health services, as well as their reasoning about personal distress and healing [31]. Similarly, the reliance on verbal rather than written communication helps to promote the participation of participants with serious and enduring mental health problems [31, 41]. Although FGD has several important outcomes, there are some limitations that need critical consideration. Ekblad and Baarnhielm [31] for example suggest, that marginalised participants may not always feel free to talk about private issues regarding their condition at the group level mostly due to perceived stigma and group confidentiality.

Some studies reviewed recommended that attempting to capture comprehensive information and analysing group interactions in mental health research requires the research method to use field notes as a supplementary data source to help validate the FGDs [31, 40, 41]. The use of field notes in addition to FGDs essentially provides greater detail in the accounts of consumers’ subjective experiences. Furthermore, Montgomery and Bailey [40] suggest that field notes require observational sensitivity, and also require having specific content such as descriptive and interpretive data.

Three studies in this section suggested that in-depth interviews are used to collect data from consumers of mental health services [19, 30, 34]. This approach is particularly important to explore the behaviour, subjective experiences and psychological processes; opinions, and perceptions of mental health services. de Jong and Van Ommeren [30] recommend that in-depth interviews help to collect data on culturally marked disorders, their personal and interpersonal significance, patient and family explanatory models, individual and family coping styles, symptom symbols and protective mediators. Palinkas [19] also highlights that the structured narrative form of extended interviewing is the type of in-depth interview used in mental health research. This approach provides participants with the opportunity to describe the experience of living with an illness and seeking services that assist them.

### Consideration for data collection

Six studies recommended consideration required in the data collection process [31, 32, 37, 41, 47, 49]. Some studies highlighted that consumers of mental health services might refuse to participate in research due to several factors [37] like the severity of their illness, stigma and discrimination [41]. Subsequently, such issues are recommended to be addressed by building confidence and trust between the researcher and consumers [31, 37]. This is a significant prerequisite, as it can sensitise and normalise the research process and aims with the participants prior to discussing their personal mental health issues. Similarly, some studies added that the researcher can gain the confidence of service providers who manage consumers of mental health services [41, 47], seek ethical approval from the relevant committee(s) [41, 47], meet and greet the consumers of mental health services before data collection, and arrange a mutually acceptable venue for the groups and possibly supply transport [41].

Two studies further suggested that the cultural and social differences of the participants need consideration [26, 31]. These factors could influence the perception and interpretation of ethical issues in the research situation.

Additionally, two studies recommended the use of standardised assessment instruments for mental health research that involve quantitative data collection [33, 49]. A recent survey suggested that measures to standardise the data collection approach can convert self-completion instruments to interviewer-completion instruments [49]. The interviewer can then read the items of the instruments to respondents and record their responses. The study further suggested the need to collect demographic and behavioural information about the participant(s).

### Preparing for data collection

Eight studies highlighted the procedures involved in preparing for data collection in mental health research [25, 30, 33–35, 39, 41, 49]. These studies suggest that the preparation process involve organising meetings of researchers, colleagues and representatives of the research population. The meeting of researchers generally involves training of interviewers about the overall design, objectives and research questions associated with the study. de Jong and Van Ommeren [30] recommended that preparation for the use of quantitative data encompasses translating and adapting instruments with the aim of achieving content, semantic, concept, criterion and technical equivalence.

### Quality assurance procedures in mental health research

This section describes the quality assurance procedures used in mental health research. Quality assurance is explained according to three sub-themes: 1) seeking informed consent, 2) the procedure for ensuring quality assurance in a quantitative study and 3) the procedure for ensuring quality control in a qualitative study. The sub-themes are explained in the following content.

### Seeking informed consent

The papers analysed for the integrative review suggested that the rights of participants to safeguard their integrity must always be respected, and so each potential subject must be adequately informed of the aims, methods, anticipated benefits and potential hazards of the study and any potential discomforts (see Table 3). Seven studies highlight that potential participants of mental health research must be consented to the study prior to data collection [25, 26, 33, 35, 37, 39, 47]. The consent process helps to assure participants of anonymity and confidentiality and further explain the research procedure to them. Baarnhielm and Ekblad [26] argue that the research should be guided by four basic moral values for medical ethics, autonomy, non-maleficence, beneficence, and justice. In particular, potential consumers of mental health services who may have severe conditions and unable to consent themselves are expected to have their consent signed by a respective family caregiver [37]. Latvala, Vuokila-Oikkonen [37] further suggested that researchers are responsible to agree on the criteria to determine the competency of potential participants in mental health research. The criteria are particularly relevant when potential participants have difficulties in understanding information due to their mental illness.

### Procedure for ensuring quality control (quantitative)

Several studies highlighted procedures for ensuring quality control in mental health research (see Table 3). The quality control measures are used to achieve the highest reliability, validity and timeliness. Some studies demonstrate that ensuring quality control should consider factors such as pre-testing tools [25, 49], minimising non-response rates [25, 39] and monitoring of data collection processes [25, 33, 49].

Accordingly, two studies suggested that efforts should be made to re-approach participants who initially refuse to participate in the study. For instance, Liu, Huang [39] recommended that when a consumer of mental health services refuse to participate in a study (due to low self-esteem) when approached for the first time, a different interviewer can re-approach the same participant to see if they are more comfortable to participate after the first invitation. Three studies further recommend that monitoring data quality can be accomplished through “checks across individuals, completion status and checks across variables” [25, 33, 49]. For example, Alonso, Angermeyer [25] advocate that various checks are used to verify completion of the interview, and consistency across instruments against the standard procedure.

### Procedure for ensuring quality control (qualitative)

Four studies highlighted the procedures for ensuring quality control of qualitative data in mental health research [19, 32, 37, 46]. A further two studies suggested that the quality of qualitative research is governed by the principles of credibility, dependability, transferability, reflexivity, confirmability [19, 32]. Some studies explain that the credibility or trustworthiness of qualitative research in mental health is determined by methodological and interpretive rigour of the phenomenon being investigated [32, 37]. Consequently, Fossey, Harvey [32] propose that the methodological rigour for assessing the credibility of qualitative research are congruence, responsiveness or sensitivity to social context, appropriateness (importance and impact), adequacy and transparency. Similarly, interpretive rigour is classified as authenticity, coherence, reciprocity, typicality and permeability of the researcher’s intentions; including engagement and interpretation [32].

Robinson [46] explained that transparency (openness and honesty) is achieved if the research report explicitly addresses how the sampling, data collection, analysis, and presentation are met. In particular, efforts to address these methodological issues highlight the extent to which the criteria for quality profoundly interacts with standards for ethics. Similarly, responsiveness, or sensitivity, helps to situate or locate the study within a place, a time and a meaningful group [46]. The study should also consider the researcher’s background, location and connection to the study setting, particularly in the recruitment process. This is often described as role conflict or research bias.

In the interpretive phenomenon, coherence highlights the ability to select an appropriate sampling procedure that mutually matches the research aims, questions, data collection, analysis, as well as any theoretical concepts or frameworks [32, 46]. Similarly, authenticity explains the appropriate representation of participants’ perspectives in the research process and the interpretation of results. Authenticity is maximised by providing evidence that participants are adequately represented in the interpretive process, or provided an opportunity to give feedback on the researcher’s interpretation [32]. Again, the contribution of the researcher’s perspective to the interpretation enhances permeability. Fossey, Harvey [32] further suggest that reflexive reporting, which distinguishes the participants’ voices from that of the researcher in the report, enhances the permeability of the researcher’s role and perspective.

One study highlighted the approaches used to ensure validity in qualitative research, which includes saturation, identification of deviant or non-confirmatory cases, member checking and coding by consensus. Saturation involves completeness in the research process, where all relevant data collection, codes and themes required to answer the phenomenon of inquiry are achieved; and no new data emerges [19]. Similarly, member checking is the process whereby participants or others who share similar characteristics review study findings to elaborate on confirming them [19]. The coding by consensus involves a collaborative approach to analysing the data. Ensuring regular meetings among coders to discuss procedures for assigning codes to segments of data and resolve differences in coding procedures, and by comparison of codes assigned on selected transcripts to calculate a percentage agreement or kappa measure of interrater reliability, are commonly applied [19].

Two studies recommend the need to acknowledge the importance of generalisability (transferability). This concept aims to provide sufficient information about the research setting, findings and interpretations for readers to appropriately determine the replicability of the findings from one context, or population to another, otherwise known as reliability in quantitative research [19, 32]. Similarly, the researchers should employ reflexivity as a means of identifying and addressing potential biases in data collection and interpretation. Palinkas [19] suggests that such bias is associated with theoretical orientations; pre-conceived beliefs, assumptions, and demographic characteristics; and familiarity and experience with the methods and phenomenon. Another approach to enhance the rigour of analysis involves peer debriefing and support meetings held among team members which facilitate detailed auditing during data analysis [19].

## Discussion

The integrative review was conducted to synthesise evidence into recommended methodological considerations when conducting mental health research. The evidence from the review has been discussed according to five major themes: 1) mixed methods study in mental health research; 2) qualitative study in mental health research; 3) sampling in mental health research; 4) data collection in mental health research; and 5) quality assurance procedures in mental health research.

### Mixed methods study in mental health research

The evidence suggests that mixed methods approach in mental health are generally categorised according to their function (rationale, objectives or purpose), structure and process [18, 19, 43, 48]. The mixed methods study can be conducted for the purpose of achieving convergence, complementarity, expansion, development and sampling [18, 19, 43]. Researchers conducting mental health studies should understand the underlying functions or purpose of mixed methods. Similarly, mixed methods in mental health studies can be structured simultaneously (concurrent) and sequential [18, 19, 42, 43, 48]. More importantly, the process of combining qualitative and quantitative data can be achieved through merging or converging, connecting and embedding one data set within the other [18, 19, 42, 43, 48]. The evidence further recommends that researchers need to understand the stage of integrating the two sets of data and the rationale for doing so. This can inform researchers regarding the best stage and appropriate ways of combining the two components of data to adequately address the research question(s).

The evidence recommended some methodological consideration in the design of mixed methods projects in mental health [18, 19, 45]. These issues include establishing early collaboration, becoming familiar with the topic, sharing information on the topic, negotiating any emerging problems and seeking contributions from team members. The involvement of various expertise could ensure that methodological issues are clearly identified. However, addressing such issues midway, or late through the design can negatively affect the implementation [45]. Any robust discoveries can rarely be accommodated under the existing design. Therefore, the inclusion of various methodological expertise during inception can lead to a more robust mixed-methods design which maximises the contributions of team members. Whilst fundamental and philosophical differences in qualitative and quantitative methods may not be resolved, some workable solutions can be employed, particularly if challenges are viewed as philosophical rather than personal [45]. The cultural issues can be alleviated by understanding the concepts, norms and values of the setting, further to respecting and including perspectives of the various stakeholders.

### Qualitative study in mental health research

The review findings suggest that qualitative methods are relevant when conducting mental health research. The qualitative methods are mostly used where there has been limited previous research and an absence of theoretical perspectives. The approach is also used to gather initial pilot data. More importantly, the qualitative methods are relevant when we want to understand sensitive issues, especially from consumers of mental health services, where the ‘lived experience is paramount [19, 28, 44]. Qualitative methods can help understand the experiences of consumers in the process of treatment, as well as their therapeutic relationship with mental health professionals. The experiences of consumers from qualitative data are particularly important in developing clinical policy [28]. The review findings find two paradigms of qualitative methods are used in mental health research. These paradigms are the interpretive and critical approach [32]. The interpretive qualitative method(s) include phenomenology, ethnography and narrative approaches [32, 36]. Conversely, critical qualitative approaches are participatory action research and emancipatory approach. The review findings suggest that these approaches to qualitative methods need critical considerations, particularly when dealing with consumers of mental health services.

### Sampling in mental health research

The review findings identified several sampling techniques used in mental health research. Quantitative studies, usually employ probability sampling, whilst qualitative studies use non-probability sampling [25, 34]. The most common sampling techniques for quantitative studies are multi-stage sampling, which involves systematic, stratified, random sampling and consecutive sampling. In contrast, the predominant sampling approaches for qualitative studies are purposive [19, 28, 32, 42, 46], snowballing [30, 32, 46] and theoretical sampling [31, 32].

The sampling of consumers of mental health services requires some important considerations. The sampling should consider the appropriateness and adequacy of the sampling approach, diversity and variety of consumers of services, attributes such as social, or cultural experiences, shared concerns related to the study, practical and organisational skills, as well as ethical and sensitivity issues are all relevant [31, 32, 46]. Sampling consumers of mental health services should also consider the homogeneity and heterogeneity of consumers. However, failure to address these considerations can present difficulty in sampling and subsequently result in selection and reporting bias in mental health research.

### Data collection in mental health research

The evidence recommends several data collection approaches in collecting data in mental health research, including focus group discussion, extended in-depth interviews, observations, field notes, Delphi data collection and quasi-statistical techniques. The focus group discussions appear as an approach widely used to collect data from consumers of mental health services [19, 28, 30, 31, 41, 44, 47]. The focus group discussion appears to be a significant source of obtaining information. This approach promotes the participation of consumers with severe conditions, particularly at the group level interaction. Mental health researchers are encouraged to use this approach to collect data from consumers, in order to promote group level interaction. Additionally, field notes can be used to supplement information and to more deeply analyse the interactions of consumers of mental health services. Field notes are significant when wanting to gather detailed accounts about the subjective experiences of consumers of mental health services [40]. Field notes can help researchers to capture the gestures and opinions of consumers of mental health services which cannot be covered in the audio-tape recording. Particularly, the field note is relevant to complement the richness of information collected through focus group discussion from consumers of mental health services.

Furthermore, it was found that in-depth interviews can be used to explore specific mental health issues, particularly culturally marked disorders, their personal and interpersonal significance, patient and family explanatory models, individual and family coping styles, as well as symptom symbols and protective mediators [19, 30, 34]. The in-depth interviews are particularly relevant if the study is interested in the lived experiences of consumers without the contamination of others in a group situation. The in-depth interviews are relevant when consumers of mental health services are uncomfortable in disclosing their confidential information in front of others [31]. The lived experience in a phenomenological context preferably allows the consumer the opportunity to express themselves anonymously without any tacit coercion created by a group context.

The review findings recommend significant factors requiring consideration when collecting data in mental health research. These considerations include building confidence and trust between the researcher and consumers [31, 37], gaining confidence of mental health professionals who manage consumers of mental health services, seeking ethical approval from the relevant committees, meeting consumers of services before data collection as well as arranging a mutually acceptable venue for the groups and providing transport services [41, 47]. The evidence confirms that the identification of consumers of mental health services to participate in research can be facilitated by mental health professionals. Similarly, the cultural and social differences of the consumers of mental health services need consideration when collecting data from them [26, 31].

Moreover, our review advocates that standardised assessment instruments can be used to collect data from consumers of mental health services, particularly in quantitative data. The self-completion instruments for collecting such information can be converted to interviewer-completion instruments [33, 49]. The interviewer can read the questions to consumers of mental health services and record their responses. It is recommended that collecting data from consumers of mental health services requires significant preparation, such as training with co-investigators and representatives from consumers of mental health services [25, 30, 33–35, 39, 49]. The training helps interviewers and other investigators to understand the research project, particularly translating and adapting an instrument for the study setting with the aim to achieve content, semantic, concept, criteria and technical equivalence [30]. The evidence indicates that there is a need to adequately train interviewers when preparing for fieldwork to collect data from consumers of mental health services.

### Quality assurance procedures in mental health research

The evidence provides several approaches that can be employed to ensure quality assurance in mental health research involving quantitative methods. The quality assurance approach encompasses seeking informed consent from consumers of mental health services [26, 37], pre-testing of tools [25, 49], minimising non-response rates and monitoring of the data collection process [25, 33, 49]. The quality assurance process in mental health research primarily aims to achieve the highest reliability, validity and timeliness, to improve the quality of care provided. For instance, the informed consent exposes consumers of mental health services to the aim(s), methods, anticipated benefits and potential hazards and discomforts of participating in the study. Herein, consumers of mental health services who cannot respond to the inform consent process because of the severity of their illness can have it signed by their family caregivers. The implication is that researchers should determine which category of consumers of mental health services need family caregivers involved in the consent process [37].

The review findings advises that researchers should use pre-testing to evaluate the data collection procedure on a small scale and then to subsequently make any necessary changes [25]. The pre-testing aims to help the interviewers get acquainted with the procedures and to detect any potential problems [49]. The researchers can discuss the findings of the pre-testing and then further resolve any challenges that may arise prior to the actual field work being commenced. The non-response rates in mental health research can be minimised by re-approaching consumers of mental health services who initially refuse to participate in the study.

In addition, quality assurance for qualitative data can be ensured by applying the principles of credibility, dependability, transferability, reflexivity, confirmability [19, 32]. It was found that the credibility of qualitative research in mental health is achieved through methodological and interpretive rigour [32, 37]. The methodological rigour for assessing credibility relates to congruence, responsiveness or sensitivity to a social context, appropriateness, adequacy and transparency. By contrast, ensuring interpretive rigour is achieved through authenticity, coherence, reciprocity, typicality and permeability of researchers’ intentions, engagement and interpretation [32, 46].

### Strengths and limitations

The evidence has several strengths and limitations that require interpretation and explanation. Firstly, we employed a systematic approach involving five stages of problem identification, literature search, data evaluation, data synthesis and presentation of results [21]. Similarly, we searched six databases and developed a data extraction form to extract information. The rigorous process employed in this study, for instance, searching databases and data extraction forms, helped to capture comprehensive information on the subject.

The integrative review has several limitations largely related to the search words, language limitations, time period and appraisal of methodological quality of included papers. In particular, the differences in key terms and words concerning methodological issues in the context of mental health research across cultures and organisational contexts may possibly have missed some relevant articles pertaining to the study. Similarly, limiting included studies to only English language articles and those published from January 2000 to July 2018 could have missed useful articles published in other languages and those published prior to 2000. The review did not assess the methodological quality of included papers using a critical appraisal tool, however, the combination of clearly articulated search methods, consultation with the research librarian, and reviewing articles with methodological experts in mental health research helped to address the limitations.

## Conclusion

The review identified several methodological issues that need critical attention when conducting mental health research. The evidence confirms that studies that addressed methodological considerations in conducting mental health research largely focuses on qualitative studies in a transcultural setting, in addition to lessons from multi-site surveys in mental health research. Specifically, the methodological issues related to the study design, sampling, data collection processes and quality assurance are critical to the research design chosen for any particular study. The review highlighted that researchers conducting mental health research can establish early collaboration, familiarise themselves with the topic, share information on the topic, negotiate to resolve any emerging problems and seek the contribution of clinical (or researcher) team members on the ground. In addition, the recruitment of consumers of mental health services should consider the appropriateness and adequacy of sampling approaches, diversity and variety of consumers of services, their social or cultural experiences, practical and organisational skills, as well as ethical and sensitivity issues.

The evidence confirms that in an attempt to effectively recruit and collect data from consumers of mental health services, there is the need to build confidence and trust between the researcher and consumers; and to gain the confidence of mental health service providers. Furthermore, seeking ethical approval from the relevant committee, meeting with consumers of services before data collection, arranging a mutually acceptable venue for the groups, and providing transport services, are all further important considerations. The review findings establish that researchers conducting mental health research should consider several quality assurance issues. Issues such as adequate training prior to data collection, seeking informed consent from consumers of mental health services, pre-testing of tools, minimising non-response rates and monitoring of the data collection process. More specifically, quality assurance for qualitative data can be achieved by applying the principles of credibility, dependability, transferability, reflexivity, confirmability.

Based on the findings from this review, it is recommended that mental health research should adequately consider the methodological issues regarding study design, sampling, data collection procedures and quality assurance issues to effectively conduct meaningful research.

## Additional file

Additional file 1: Data extraction form. (DOCX 18 kb)

## Data Availability

Not applicable

## Abbreviations

FGDs: focus group discussions
JBI: Joanna Briggs Institute
PRISMA: Preferred Reporting Items for Systematic Reviews and Meta-Analyses
