# Supplementary material for: An integrative review on methodological considerations in mental health research – design, sampling, data collection procedure and quality assurance
Source: Arch Public Health. 2019 Oct 10;77:37. doi: 10.1186/s13690-019-0363-z (PMC6785873; doi:10.1186/s13690-019-0363-z)
Supplement: Supplementary file 1 — Data extraction form. (DOCX 18 kb) [file 13690_2019_363_MOESM1_ESM.docx]

**Appendix 1 Data extraction form**

| **Study ID** |  |
| --- | --- |
| **Study Details** |  |
| Citation |  |
| Year of publication(s) |  |
| Author(s) |  |
| Contact details of the lead author |  |
| Funder / sponsoring organisation |  |
| Publication type  Example:   - Journal article - Report (specify) - Case study - Other |  |
| Objective |  |
| **Primary subject area of the paper** |  |
| The paper may focus on one or more sectors ie   - Study design - Methods - Sampling - Data collection - Data analysis - Quality assurance - Other |  |
| **Study design** | **Please describe the type of study design? ie application of the design** |
|  | |
| **Methods** | **Please explain the type of methods? ie how to apply the method** |
|  | |
| **Sampling** | **Please describe the sampling? ie how to practically apply the sampling** |
|  | |
| **Data collection** | **Please describe the approaches used to collect data?** |
|  | |
| **Data analysis** | **Please describe the approaches to analyse data?** |
|  | |
| **Quality assurance** | **Please explain how to ensure quality assurance assessing the quality of psychiatric services?** |
|  | |
| **Please report any additional information on the methods, design, data collection, and analysis and quality assurance in assessing quality psychiatric services?** | |
|  | |
| **Recommendation – methodological gaps and contribution to the specialty** | |
|  | |
| **Identifiable references to follow up** | |
|  | |
